# Self-reported nutritional supplement usage among professional mixed martial artists – preliminary findings

**DOI:** 10.1186/1550-2783-9-S1-P18

**Published:** 2012-11-19

**Authors:** Paul La Bounty, Elfego Galvan, Jeremy Reid, Bill I Campbell, Jeremy McElroy, Eva Doyle, Tony Boucher

**Affiliations:** 1 Baylor University, Waco, TX, USA; 2 Texas A&M University, College Station, TX, USA; 3 University of South Florida Tampa, FL, USA

## Background

Although mixed martial arts (MMA) has been around for decades in other countries such as Brazil, it is still a relatively new sport for most of the world. Research on combative sport athletes has focused primarily on the various individual sports that compose MMA such as judo, boxing, and wrestling. To date, there is limited peer-reviewed research investigating professional mixed martial artists. More specifically, there is very limited information regarding the dietary supplement habits of current professional mixed martial arts fighters. Thus, the purpose of this study was to investigate various dietary habits, beliefs, and nutritional supplement usage, in professional mixed martial artists.

## Methods

Male professional mixed martial artists (18-50 y/o) in every recognized weight class (i.e., bantam weight, featherweight, lightweight, welterweight, middleweight, light-heavy weight, and heavy weight) were eligible to participate in this this study. Participants were recruited from various mixed martial art gyms primarily from, but not limited to, the states of Texas and Nevada. The investigators developed a new questionnaire that addressed various aspects of nutritional intake, sport supplement beliefs and usage, as well as weight cutting strategies. Once developed, the questionnaire was reviewed by 2 registered dietitians who have expertise in exercise nutrition, 3 exercise physiologists (2 of which are Certified Strength and Conditioning Specialists), and a physical therapist. Before the questionnaire was administered, a copy of the questionnaire was given to the participant so that they could visually read along as the questions were being asked to them by the investigators. The investigators verbally asked the participants the questions included in the questionnaire and wrote down their responses. The data presented in this abstract focuses on sport supplement usage and weight cutting in the 48 hours prior to competition. Averages and standard deviations were calculated on Microsoft Excel.

## Results

To date, 11 male professional mixed martial artists (29.9 ± 3.6 y/o; range: 23-37 y/o) participated in this ongoing study. On average, the participants have been competing professionally for 5.3 ± 4.6 years (range: ~ 0.7 – 12 years) and have had 14.2 ± 15.9 professional MMA fights (range: 2-42). Featherweight (~145 lbs), lightweight (~155 lbs), welterweight (~ 170 lbs), light heavyweight (~ 205 lbs) and heavyweight (> 205 lbs) weight classes were represented in this sample. Out of the 11 participants who completed the questionnaire, 27.3% reported that they regularly consume creatine at least five to six times per week. Beta-alanine was consumed by 36.4% of the participants at least two to four times per week. Fish oil was consumed by 63.6% of the participants at least two to four times per week, while one participant reported consuming fish oil less often than once per month. Additionally, 36.4% of the participants consumed a thermogenic supplement five to six times per week. Furthermore, hydroxyl-methylbutyrate (HMB) was not consumed by any of the respondents. Regarding weight cutting practices, the respondents lost an average of 12.73 ± 7.2 lbs. (range: 0-22 lbs) during the forty-eight hours prior to competition.

## Conclusions

The results of the study report common dietary supplements consumed by professional mixed martial artists. Current research regarding the dietary habits of professional mixed martial artists is currently lacking and thus more research is needed.

